# Fungal Associates of Soft Scale Insects (Coccomorpha: Coccidae)

**DOI:** 10.3390/cells10081922

**Published:** 2021-07-29

**Authors:** Teresa Szklarzewicz, Katarzyna Michalik, Beata Grzywacz, Małgorzata Kalandyk-Kołodziejczyk, Anna Michalik

**Affiliations:** 1 Department of Developmental Biology and Morphology of Invertebrates, Faculty of Biology, Institute of Zoology and Biomedical Research, Gronostajowa 9, 30-387 Kraków, Poland; teresa.szklarzewicz@uj.edu.pl (T.S.); katarzyna.michalik.bio@gmail.com (K.M.); 2 Institute of Systematics and Evolution of Animals, Polish Academy of Sciences, Sławkowska 17, 31-016 Kraków, Poland; grzywacz@isez.pan.krakow.pl; 3 Faculty of Natural Sciences, Institute of Biology, Biotechnology and Environmental Protection, University of Silesia, Bankowa 9, 40-007 Katowice, Poland; malgorzata.kalandyk@us.edu.pl

**Keywords:** soft scale insects, *Ophiocordyceps*, symbiosis, transovarial transmission

## Abstract

*Ophiocordyceps* fungi are commonly known as virulent, specialized entomopathogens; however, recent studies indicate that fungi belonging to the Ophiocordycypitaceae family may also reside in symbiotic interaction with their host insect. In this paper, we demonstrate that *Ophiocordyceps* fungi may be obligatory symbionts of sap-sucking hemipterans. We investigated the symbiotic systems of eight Polish species of scale insects of Coccidae family: *Parthenolecanium corni*, *Parthenolecanium fletcheri*, *Parthenolecanium pomeranicum*, *Psilococcus ruber*, *Sphaerolecanium prunasti*, *Eriopeltis festucae*, *Lecanopsis formicarum* and *Eulecanium tiliae*. Our histological, ultrastructural and molecular analyses showed that all these species host fungal symbionts in the fat body cells. Analyses of ITS2 and Beta-tubulin gene sequences, as well as fluorescence in situ hybridization, confirmed that they should all be classified to the genus *Ophiocordyceps*. The essential role of the fungal symbionts observed in the biology of the soft scale insects examined was confirmed by their transovarial transmission between generations. In this paper, the consecutive stages of fungal symbiont transmission were analyzed under TEM for the first time.

## 1. Introduction

Scale insects (coccoids) are plant sap-sucking hemipterans that are considered serious pests in agriculture, horticulture, and forestry. These insects cause direct damage to plants through sap-sucking and the injection of toxic saliva into plant tissue, which is a cause of the retardation of plant growth and recovery, and furthermore, may lead to the death of the whole or part of the plant if the infestation is severe. Scale insects are rarely known as vectors of bacterial pathogens or phytoplasmas, and only a few species are involved in virus transmission [1]. Most species of scale insects also cause indirect damage by producing a carbohydrate-rich solution, referred to as honeydew, which is a medium for the growth of saprophytic fungi known as “sooty molds”, forming black superficial colonies that also reduce the host plant photosynthesis rates, further diminishing the vigor of the plant (e.g., [1,2,3]).

Scale insects are highly diverse in terms of the morphology of their external and internal organs, reproductive strategies and chromosome systems, as well as symbiotic systems, which makes them an interesting group of insects to study [4]. After the Diaspididae and Pseudococcidae, the Coccidae (soft scales, coccids) is the third largest family of scale insects in terms of species richness. There are 1281 described species of coccids in 176 genera. Soft scales are widely distributed in all zoogeographical regions; however, they predominantly occur in the tropics and subtropics [2,5,6,7]. The Coccidae, like other scale insect families, exhibit a remarkable dimorphism. The adult females are wingless and lack a well-defined head, thorax, and abdomen. The adult males are usually winged, with distinct body parts, and do not possess functional mouthparts. A large number of soft scale species are notorious plant pests that are of great economic importance to crops. Many pests of the Coccidae have been introduced into new zoogeographical regions, thus making them cosmopolitan [2].

In several insect groups, including scale insects, a mutualistic relationship with microorganisms (bacteria or fungi) evolved. The use of genomic analyses has confirmed earlier assumptions that the occurrence of symbiotic associates in the insect body is associated with the poor diet of the host-insect, e.g., plant sap-sucking hemipterans receiving amino acids, and blood-sucking insects receiving B vitamins from their mutualists [8].

As typical phloem-feeders, scale insects live in symbiotic association with microorganisms; however, in comparison with close relatives such as aphids, whiteflies, and psyllids, these insects are characterized by highly diverse symbiotic systems. Scale insects may live in mutualistic relationships with different species of bacteria or fungal symbionts. They may have only one symbiont or several species of microorganisms. Symbionts may be harbored in the fat body cells, in the midgut epithelium, in the specialized cells of mesodermal origin termed bacteriocytes, or inside the cells of other bacteria. Scale insects also developed different modes of transmission of their symbiotic associates from mother to offspring [9,10,11,12].

In contrast to other families of scale insects, symbionts of soft scale insects have not been as extensively examined through the use of modern ultrastructural and molecular methods. The results of histological studies (reviewed in [9,10,13]) have indicated that these insects are hosts to obligate fungal symbionts that may be localized freely in hemolymph or intracellularly in fat body cells, and are transovarially inherited. Studies recently conducted with the use of molecular methods and phylogenetic analyses have allowed the identification of the symbiotic associates of seven species of the Coccidae from the Mediterranean region and 28 species from southern China as the *Ophiocordyceps*-allied fungus (phylum Ascomycota) [14,15]. It is noteworthy that, for many years, fungi belonging to the genus *Ophiocordyceps*, like other members of Ophiocordycypitaceae and Cordycypitaceae, were known mainly as entomopathogenic microorganisms [16,17]. They may attack various species of insects, e.g., ants, beetles, butterflies, and hemipterans. The hyphae of these fungi penetrate the body wall and destroy their internal tissue: fat body cells, hemocytes, muscle, nerve ganglions, and the intestine. In each case, insects infected by these fungi die before beginning their reproductive phase, i.e., within 48–96 h of penetration [16]. For this reason, entomopathogenic fungi have also been tested as biological control agents for whiteflies, lepidopterans and scale insects [18,19]. The finding of a close relationship between fungal entomopathogens and symbionts has led to the hypothesis that during their co-evolution, the interaction between both of these partners shifted from parasitism to mutualism [20,21].

The aim of this study was to further explore the symbiotic systems of the Coccidae family: (1) to determine the systematic position of symbionts, (2) to verify whether symbiosis with fungi is a general rule of this family, (3) to show whether the symbiosis is the result of the single infection of the ancestor of Coccidae or multiple independent infections, (4) to describe symbiont distribution and ultrastructure as well as a mode of transmission from mother to progeny in eight species from three subfamilies of Central European origin.

## 2. Material and Methods

### 2.1. Insects

Eight species of the Coccidae family: *Parthenolecanium corni* (Bouché, 1844), *Parthenolecanium fletcheri* (Cockerell, 1893), *Parthenolecanium pomeranicum* (Kawecki, 1954), *Eriopeltis festucae* (Boyer de Fonscolombe, 1834), *Lecanopsis formicarum* Newstead, 1893, *Sphaerolecanium prunastri* (Boyer de Fonscolombe, 1834), *Eulecanium tiliae* (Linnaeus, 1758) and *Psilococcus ruber* Borchsenius, 1952 were collected in unprotected areas in Poland between the years 2017 and 2019 from their host plants. The localities, collection dates, and host plants of the investigated species have been summarized in Table 1. Species of the Coccidae family were assigned to subfamilies according to Koteja [22].

### 2.2. Light (LM) and Electron Microscopy (TEM)

Females of the investigated species, destined for detailed histological and ultrastructural analysis, were fixed in 2.5% glutaraldehyde in 0.05 M phosphate buffer (pH 7.4) and stored in a fridge for 1–4 weeks. After this time, the samples were rinsed five times in the buffer with sucrose (5.8 g/100 mL), postfixed in buffered 1% osmium tetroxide for 2 h, and then dehydrated in an ethanol series (30%, 50%, 70%, 90%, 100%) and acetone. Finally, the samples were embedded in epoxy resin Epon 812 (Serva, Heidelberg, Germany) ([23]—modified). For the histological analyses, semithin sections (1 μm thick) were stained in 1% methylene blue in 1% borax and photographed under a Nikon Eclipse 80i light microscope. For ultrastructural analyses, ultrathin sections (90 nm thick) were doubly contrasted with uranyl acetate and lead citrate and subsequently examined and photographed under a Jeol JEM 2100 at 80 kV transmission electron microscope. The number of specimens used for histological and ultrastructural analyses is summarized in Table 1.

### 2.3. Molecular Analyses

Specimens destined for molecular analysis were fixed in 100% ethanol. In the case of the removal of the surphase’s contaminations, the specimens were placed in 5% sodium hypochlorite for 1 min and then rinsed in distilled water three times for one minute. Then the cuticle was removed, and the DNA isolated only from the fat body and internal organs. DNA extraction was performed separately from 3-7 individuals of each species (see Table 1) using the Bio-Trace DNA Purification Kit (EURx, Gdańsk, Poland) following manufacturer protocol and subsequently stored at −20 °C for further analysis.

The fungal associates of the species examined were identified and characterized based on sequences of two genes: Internal Transcribed Spacer 2 of nuclear ribosomal RNA (ITS 2) and Beta-tubulin gene using primers: ITS3/ITS4 [24] and Ophi_Btub44448F/Ophi_Btub5243R (D. Vanderpool, unpublished), respectively. The mitochondrial cytochrome c oxidase subunit I (COI) gene of soft scale insects was amplified using the primer pair PCoF1 and HCO [25]. The conditions for all the PCR reactions were an initial denaturation step at 94 °C for 5 min, followed by 33 cycles at 94 °C for 30 s, Tm for 40 s, 70 °C for 1 min 40 s and a final extension step of 5 min at 72 °C. The PCR products were visualized on 1.5% agarose gel stained with Simply Safe (EURx, Gdańsk, Poland), purified with the Gel-Out Concentrator (A&A Biotechnology, Gdańsk, Poland) kit following manufacturer protocol, and subsequently sequenced. The sequences of the primers have been listed in Table S1. The nucleotide sequences obtained were deposited in the GenBank database under the accession numbers: ITS (MN733271-MN733277, MZ594469); COI (MN603157, MN603159-MN603160, MN603162-MN603164, MZ567176), Beta-tubulin (MN750822-MN750828, MZ576194).

### 2.4. Fluorescence In Situ Hybridization (FISH)

Fluorescence in situ hybridization (FISH) was conducted with the probe Hyp760 specific for the 18S rRNA gene of *Ophiocordyceps* fungi [26] (Table S1). Two individuals of each species that were preserved in 100% ethanol were rehydrated, fixed in 4% formaldehyde for two hours and dehydrated through incubation in 80%, 90% and 100% ethanol and acetone. The material was then embedded in Technovit 8100 (Kulzer, Werheim, Germany) resin and subsequently cut into sections. Hybridization was performed using a hybridization buffer containing: 1 mL 1 M Tris-HCl (pH 8.0), 9 mL 5 M NaCl, 25 μL 20% SDS, 15 mL 30% formamide and about 15 mL of distilled water. The slides were incubated in 200 μL of hybridization solution (hybridization buffer + probes) overnight at room temperature [27]. Following this, the slides were washed in PBS three times for 10 min, then dried and covered with a ProLong Gold Antifade Reagent (Life Technologies, Carlsbad, CA, USA). The hybridized slides were then examined using a confocal laser scanning microscope Zeiss Axio Observer LSM 710.

### 2.5. Phylogenetic and Co-Phylogenetic Analyses

The sequences were aligned in a CodonCode Aligner v.8.0 (CodonCode Corporation, www.codoncode.com, 8 March 2018). Coding regions were translated to amino acids using Mega v. X [28] in order to detect frameshift mutations and internal stop codons. The Akaike Information Criteria (AIC) in MrModeltest v. 2.2 [29] was used to estimate the best-fit substitution models. Phylogenetic trees were constructed using Bayesian inference (BI) in MrBayes v. 3.2.6 [30]. For phylogenetic trees of both scale insects and fungal symbionts, MrBayes was run for six million generations, sampling every 100 generations in order to ensure the independence of the samples. Two independent runs were performed to ensure that convergence on the same posterior distribution was reached, and if the final trees converged on the same topology. The statistical confidence in the nodes was evaluated using posterior probabilities.

Co-phylogenetic and host-switching events were tested in Jane v.4 [31] using the BI host and fungal trees as input. The analysis was performed with 100 generations, population sizes of 100 and a default event–cost scheme including “co-speciation”, “duplication”, “host switch”, “losses”, and “failure to diverge”.

## 3. Results

### 3.1. Fungi Belonging to the Ophiocordycypitaceae Family Are Symbionts of the Soft Scale Insects Examined

Our histological, ultrastructural, and molecular analyses showed that all the species examined were associated with symbiotic fungi. Molecular analyses based on sequences of ITS2 and Beta-tubulin genes revealed that in all the species examined, the symbiotic fungi belonged to the Ophiocordycypitaceae family within the Ascomycota phylum (Ascomycota: Sordariomycetes: Hypocreales: Ophiocordycipitaceae).

**Figure 1:**
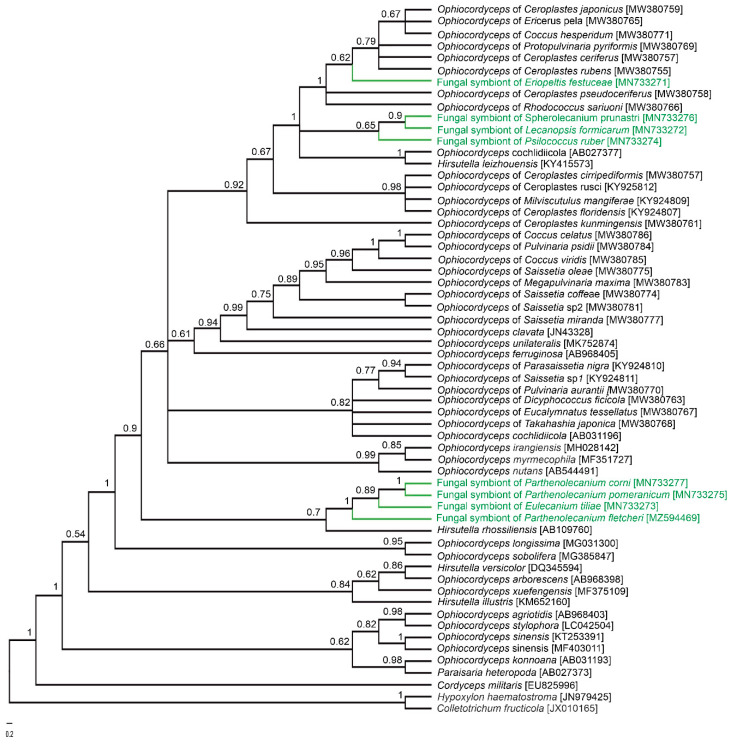
Phylogenetic tree showing the relationships between fungal symbionts of soft scale insect species, pathogenic, as well as free living fungi (constructed on the base of ITS2 genes). The numbers near the nodes refer to the Bayesian posterior probability.

**Figure 2:**
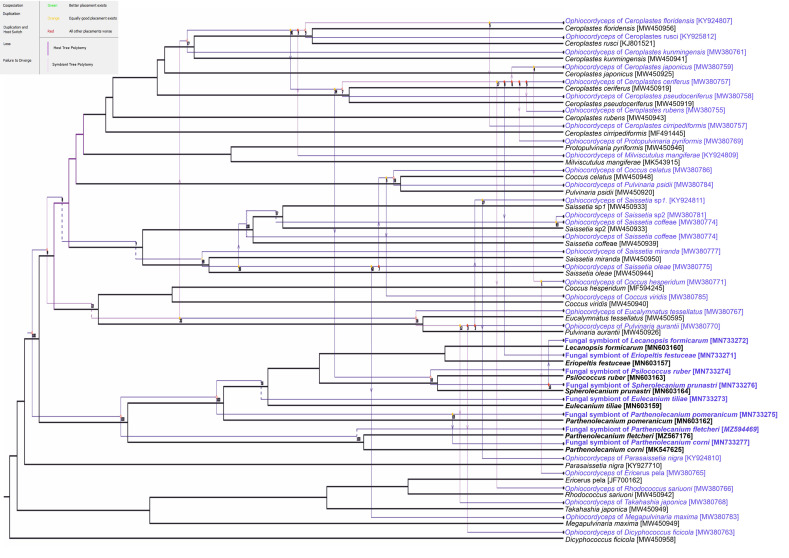
Co-phylogenetic analysis between the *Ophiocordyceps* symbionts’ tree and their host’s tree (constructed on the base of the ITS2 genes of fungal symbionts and COI genes of host scale insects). Black and blue lines indicate the phylogenies of the scale insects and *Ophiocordyceps,* respectively. Hollow red circles indicate co-speciation events, solid red and yellow circles indicate duplications, arrows indicate host events, dashed lines indicate losses, and uneven lines indicate failures to diverge.

Based on the sequences of the Beta-tubulin gene, two groups of symbiotic microorganisms may be distinguished: the first one includes symbionts of *Eulecanium tiliae*, *Parthenolecanium corni*, *Parthenolecanium pomeranicum*, and *Parthenolecanium fletcheri*, and the second one is comprised of the symbionts of remaining species. These latter sequences are almost identical (99%) and differ from the sequences in the first group by 4%. In turn, the similarity of the ITS2 sequences, which are more species-specific, ranges from 87% to 96%. Blast searches for all of the sequences obtained demonstrated the highest similarity to sequences of homologue genes of various species of *Ophiocordyceps* or its anamorphic (i.e., asexual) form—*Hirsutella*. Phylogenetic analyses also confirmed the systematic affiliation of *Hirsutella* with the genus *Ophiocordyceps*, and showed that they create a sister group, i.e., *O. cochlidiicola*, that is closely related to *H. leizhouensis*, while *O. arborescens*, *H. versilor*, and *O. xuefengensis* are closely related to *H. illustris* (Figure 1 and Figure S1). The co-phylogenetic analysis based on the ITS2 gene of fungal symbionts and the COI genes of host scale insects returned 11 potential co-speciation events, one duplication, 22 duplications with host switch, five losses, and one failure to diverge (Figure 2).

**Figure 3:**
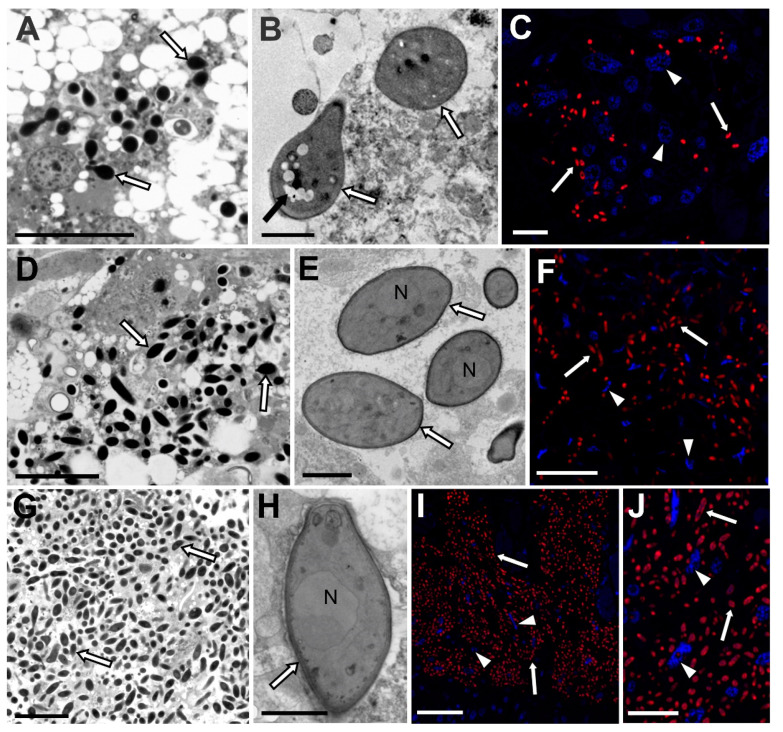
Distribution of symbiotic fungi in the body cavity of species examined (**A**–**J**). *Ophiocordyceps* fungi in the cytoplasm of the fat body cells (**A**,**D**,**G**). Light microscope, scale bar = 20 µm (**B**,**E**,**H**). TEM, scale bar = 2 µm (**C**,**F**,**I**,**J**). Confocal microscope, scale bar = 20 µm (**A**–**C**). Eriopelitnae subfamily (**A**). *Eriopeltis festucae* (**B**,**C**). *Lecanopsis formicarum* (**D**–**F**). Coccinae subfamily (**D**,**E**). *Parthenolecanium corni* (**F**). *Parthenolecanium pomeranicum* (**G**–**J**). Filippinae subfamily, *Sphaerolecanium prunastri*. N—nucleus of the fungal cell; white arrow—fungal symbiont, white arrowhead—nucleus of the fat body cell, black arrow—vacuole.

The analysis of serial semithin sections has shown that symbiotic fungi are distributed only within the fat body cells (Figure 3). They were not observed in any other tissue except the ovaries, which is related to the transovarial transmission of these symbionts between generations (see the Results subsection, which follows). The number of symbionts and their density in the host insect body are subfamily specific and are not dependent on the stage of the insect’s development (Figure 3). We observed the same amount of cells of fungi in the body cavities of larvae and mature females. The smallest number of symbionts was observed in the representatives of the Eriopeltinae subfamily (with the exception of *Psilococcus ruber*), where only single groups of fungi occur (Figure 3A–C). The highest density was observed in the members of Filippinae subfamily (Figure 3G–J). In all representatives of the Filippinae subfamily examined: *Sphaerolecanium prunasti* and *Eulecanium tiliae*, all cells of the fat body are filled with numerous symbiotic fungi (Figure 3G–J). The ultrastructural analyses showed that the cells of fungi are surrounded by a thick cell wall (Figure 3B,E,H). Large nuclei (Figure 3E,H) and vacuoles (Figure 3B) are visible in their cytoplasm.

The presence of *Ophiocodyceps* fungi in the body cavity of the soft scale insects examined was also confirmed by fluorescence in situ hybridization using an *Ophiocordyceps*-specific probe (Figure 3C,F,I,J). The microscopic observations did not show any damage to the insects’ tissue caused by fungi.

### 3.2. Fungal Associates of Soft Scale Insects Are Transovarially Transmitted between Generations

Microscopic observations revealed that fungal symbionts residing in the examined species of soft scale insects were inherited transovarially, i.e., they infect female germ cells.

**Figure 4:**
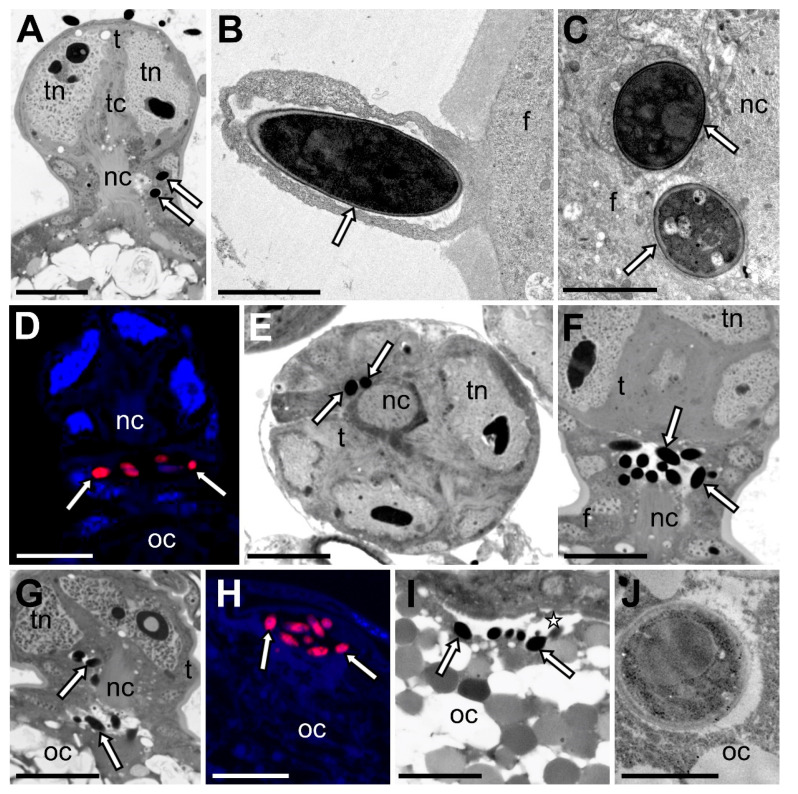
Consecutive stages of symbiont transmission between generations (**A**,**B**). Longitudinal section through the ovariole of *Parthenolecanium fletcheri*. Symbiotic fungi (white arrows) invade the follicular cells in the neck region of the ovariole (**A**). Light microscope, scale bar = 20 µm (**B**). TEM, scale bar = 2 µm (**C**,**D**). Symbiotic fungi in the cytoplasm of follicular cells of *Parthenolecanium pomeranicum* (**C**) and *Eulecanium tiliae* (**D**). (**C**) TEM, scale bar = 2 µm (**D**). Confocal microscope, scale bar = 20 µm (**E**,**F**). Symbiotic fungi surrounding the nutritive cord (**E**). *Parthenolecanium pomeranicum* (**F**). *Parthenolecanium fletcheri* (**E**,**F**). Light microscope, scale bar—20 µm (**G**). Symbiotic fungi move along the nutritive cord to the perivitelline space of *Parthenolecanium fletcheri*. Light microscope, scale bar = 20 µm (**H**). Symbiotic fungi gather in the invagination of the perivitelline space of *Eulecanium tiliae*. Confocal microscope, scale bar = 20 µm (**I**). Migration of symbionts from the perivitelline space to the oocyte cytoplasm of *Psilococcus ruber*. Light microscope, scale bar = 20 µm (**J**). Symbiotic microorganism in the oocyte cytoplasm of *Psilococcus ruber.* TEM, scale bar = 2 µm. f—follicular cell, nc—nutritive cord; oc—oocyte, t—trophocyte, tc—trophic core, tn—trophocyte nucleus, white arrow—fungal symbiont; asterisk—perivitelline space.

The ovaries of soft scale insects are composed of numerous short telotrophic ovarioles, which are subdivided into an anterior tropharium (trophic chamber) and posterior vitellarium (Figure 4A) (for further details concerning the organization of the ovaries of scale insects, see [32]). The vitellarium houses a single oocyte, which is connected with the tropharium by means of a broad nutritive cord (Figure 4A). The oocyte is surrounded by a single-layered follicular epithelium (Figure 4A). At the time the ovarioles contain the oocytes in the stage of advanced choriogenesis (Figure 4A), the fungal symbionts begin to enter follicular cells surrounding the neck region of the ovariole (i.e., the region between the tropharium and the developing oocyte) (Figure 4B). After crossing through the cytoplasm of the follicular cells (Figure 4A,C,D), symbionts temporarily gather around the nutritive cord (Figure 4E,F). Next, symbiotic microorganisms migrate along the nutritive cord to the space between follicular epithelium and oocyte surface (Figure 4G,H). Finally, symbionts enter the oocyte cytoplasm (Figure 4I,J), where they remain until the beginning of embryonic development.

## 4. Discussion

The Cordycypitaceae and Ophiocordycypitaceae families of Ascomycota include several genera that are commonly occurring entomopathogens, such as *Ophiocordyceps*, *Cordyceps*, *Hirsutella*, *Lecanicilium*, and *Metarhizium* [33]. Among them, the *Ophiocordyceps* species, which usually attack ants, are best known for their ability to manipulate ant behavior. However, recent research that applies the use of molecular techniques, and concerns the interactions of insects and various microorganisms indicates that the fungi of Ascomycota phylum may also live in symbiotic associations with insects [14,26,34]. So far, fungal associates have been found and described in many representatives of Hemiptera, Coleoptera, Diptera, and Hymenoptera, as well as in some members of Isoptera, Neuroptera, and Lepidoptera; for a review, see [35]. They belong to various families of Ascomycota; however, hemipterans are usually associated with fungi from the Ophiocordycypitaceae family.

Within the Hemiptera: Auchenorrhyncha (i.e., Fulgoromorpha (planthoppers) and Cicadomorpha (leafhoppers, treehoppers, spittlebugs, and cicadas)), the presence of *Ophiocordyceps*-allied symbionts was confirmed in some leafhoppers from the Deltocephalinae subfamily [36,37] and planthoppers from the Flatidae and Delphacidae families [38,39,40], as well as in Japanese cicadas [26]. In Hemiptera: Sternorrhyncha, the symbiosis with fungi is not as common as in Auchenorrhyncha, and has so far only been observed in some of the Hormaphidinae aphids and members of the Coccidae, Dactylopiidae, and Kermesidae families of scale insects [14,15,34,36,41,42,43].

In this paper, using microscopic and molecular techniques, we investigated symbiotic systems of soft scale insects (Coccidae) belonging to three subfamilies: Coccinae, Eriopeltinae and Filippinae. Our analyses revealed that all of the species investigated were only host to fungal symbionts. Analyses of sequences of ITS2 as well as Beta-tubulin genes demonstrated that these microorganisms are representatives of the Ophiocordycypitaceae family. However, the ITS sequences, which are more species-specific than the Beta-tubulin gene, display about 87–95% similarity to each other. Fungal symbionts of members of the Coccidae family have previously been studied by Gomez-Polo and co-workers [14] and Deng and co-workers [15]. These authors investigated seven coccid species from the Ceroplastinae and Coccinae subfamilies collected in Spain, Israel, and Cyprus. Based on a high-throughput sequencing of ribosomal genes, these authors showed that the species analyzed were mainly associated with *Ophiocordyceps* fungi.

It is believed that the occurrence of fungal associates in some auchenorrhynchans and some aphids is a result of symbiont replacement [26,36,37], e.g., in deltocephalinae leafhoppers as well as in Japanese cicadas, fungi replaced the bacteria *Nasuia* and *Hodgkinia* (respectively), in Delphacidae and Flatidae planthoppers – bacteria *Sulcia* and *Vidania,* in the aphids of Hormaphidinae subfamily–bacteria *Buchnera* [26,36,37,41,44,45,46,47]. Our phylogenetic analyses showed that *Ophiocordyceps*-allied fungi in the species of soft scale insects examined form a clade (see Figure 1), which suggests that symbiosis between these insects and their microorganisms is the result of a single infection of the ancestor of extant coccids. However, the differences observed in ITS2 sequences (5–13%) may indicate their independent evolution after the initial infection.

Since symbiotic fungi have recently been observed in various hemipteran lineages, researchers continually ask themselves about the origin of these associations [14,20,34,35,40]. The literature data indicate three possible evolutionary scenarios: (1) symbiotic fungi may derive from entomopathogenic fungi; (2) they may be the descendants of nonpathogenic commensals; or (3) the ancestor of fungal symbionts may be phytopathogenic fungi [21,35,38]. Most hemipterans are plant sap-sucking insects, and due to their mode of feeding, they are also vectors of plant pathogens. Therefore, it seems probable that they may acquire fungal symbionts from the host plant. However, the results of molecular phylogenetic analyses that indicate the close relationship between fungal symbionts of hemipterans with entomopathogenic fungi favor the concept that the ancestors of the present fungal symbionts were entomopathogens that lost their virulence and shifted to a symbiotic lifestyle. The genomic analysis of the fungal symbiont of the planthopper *Nilaparvata lugens* showed that it possesses a smaller genome than its free-living relatives and does not possess genes encoding enzymes responsible for penetrating the insect’s cuticle, solubilizing its tissue, or genes related to sexual reproduction [40].

*Ophiocordyceps* fungi, in the soft scale insects examined, are localized in the cytoplasm of the fat body cells. The same localization of the fungal symbiont was observed in other Coccidae species that have previously been examined, in the scale insect *Kermes quercus* (Kermesidae) and leafhoppers *Fieberiella septentrionalis*, *Graphocraerus ventralis* and *Orientus ishidae* [9,14,34,37]. In the Deltocephalinae leafhopper *Cicadula quadrinotata*, fungal symbionts were found to be present in the cytoplasm of midgut epithelium cells, in fat body cells, and free in the hemolymph [37]. In contrast, in Japanese cicadas and planthoppers from the Flatidae and Delphacidae families, they are harbored in the cells of a specialized host’s organs, termed mycetomes [26,47]. It seems probable that, similarly to the case of the bacterial associates of insects [48], the occurrence of fungal symbionts in the digestive tract represents the initial state of colonization of the insect body through microorganisms, in the hemolymph and in fat body cells represents the next (i.e., intermediate) stage, whereas their presence in the cells of mycetomes represents the most advanced condition of this association. It is worth mentioning that the occurrence in the fat body and in the specialized host’s cells seems to be characteristic to fungi from the Ophiocordycypitaceae family, whereas other species of fungi found so far in insects are usually localized in a different part of the digestive tract [35].

Scale insects, like other insects living in a mutualistic relationship with microorganisms, develop stable mechanisms of transmission of these associates from one generation to the next. The results of numerous studies conducted both earlier and more recently indicate that scale insects are not only characterized by diverse species and distributions of symbionts, but also by different modes of transmission to their progeny ([9,12,49,50,51,52,53,54,55,56,57,58], this study). It should be stressed that even members of the same family may inherit symbionts differently (for further details, see [58]). It should be stressed that scale insects (i.e., all the Pseudococcidae, Eriococcidae, Coccidae, and Putoidae examined so far) are unique in that they are the only group of insects in which microorganisms invade the neck region of the ovariole ([50,55,56,57], this study). Until now, the course of transmission of fungal symbionts in soft scale insects had not been studied under TEM; however, Gomez-Polo and co-workers [14] reported their presence in eggs, and thus proved that these microorganisms are transported between generations transovarially. Our observations of the ovaries of the members of the Coccidae family showed that their fungal associates are transmitted between generations similarly to bacterial symbionts in Pseudococcidae, Eriococcidae, and Putoidae [50,53,55,56,57]. One noteworthy aspect is that, just as in the case of the infestation of ovarioles through bacterial symbionts, the time of transmission of the fungal symbionts in soft scale insects is correlated with the stage of oogenesis—the microorganisms commence the infection of the ovarioles that contain the oocytes in the stage of advanced choriogenesis. Similarly to Pseudococcidae, Eriococcidae, and Putoidae, in Coccidae, symbionts invade follicular cells surrounding the nutritive cord, because this area is the only place on the oocyte surface that is devoid of egg envelopes (see Figure 4A). After the degeneration of the nutritive cord, the symbionts may enter the oocyte cytoplasm.

Numerous molecular analyses have confirmed that bacterial symbionts co-evolve with their host insects [59,60,61]. The co-diversification of fungal associates and insects has previously not been tested intensively. Our co-phylogenetic analysis, through the use of Jane software, indicated that not all the species of soft scale insects examined co-evolved with their host (see Figure 2). It is a noteworthy fact that Gomez-Polo and co-workers [14] additionally revealed that co-phylogeny of Coccidae, which they tested, and their *Ophiocordyceps* symbionts were incongruent. These authors suggested that this incongruence may be a result of the independent acquisition of fungi by particular members of Coccidae. However, taking into account the fact that some of the species examined co-evolved with their symbiotic partners (this study), it may be speculated that incongruence in the co-phylogeny of some coccids and their fungal associates may result from the independent evolution of fungal symbionts or their replacement during the evolutionary history of different species.

## Figures and Tables

**Table 1:** List of investigated species with collection details and number of individuals used in each method applied.

Species	Subfamily	Place of Collection/Locality Coordinates	Date of Collection	Host Plant	No. of Individuals Examined Using Microscopic Techniques		No. of Individuals Examined Using Molecular Techniques	
					LM	TEM	PCR	FISH
*Parthenolecanium corni*	Coccinae	Katowice 50.245638 19.007992	V, VI 2017	*Tilia cordata*	10	2	3	1
*Parthenolecanium fletcheri*		Katowice, 50.245621 19.005127 Olsztyn 50.753157 19.277790	V 2018; V 2019	*Thuja* sp.	6	4	3	1
*Parthenolecanium pomeranicum*		Katowice 50.243978 19.001704	V, VI 2017; VI 2018	*Taxus baccata*	10	2	7	3
*Eriopeltis festucae*	Eriopeltinae	Olsztyn 50.749359 19.272956	VII 2017; VI 2018	*Calamagrostis epigejos*	5	2	3	1
*Lecanopsis formicarum*		Mikoszewo 54.341908 18.992342	VIII 2018	*Festuca ovina*	5	2	7	3
*Psilococcus ruber*		Mikoszewo 54.344519 18.978498	VIII 2018	*Carex* sp.	10	4	7	3
*Sphaerolecanium prunastri*	Filippinae	Bukowno 50.274843 19.436405	VI 2017; IV 2018	*Prunus spinosa*	15	3	7	3
*Eulecanium tiliae*		Olsztyn 50.749837 19.274678	V 2018	*Tilia cordata*	6	2	3	1

## Data Availability

The data presented in this study are available on request from the corresponding author.

## Supplementary Materials

The following are available online at https://www.mdpi.com/article/10.3390/cells10081922/s1. Figure S1. Phylogenetic tree showing the relationships between fungal symbionts of soft scale insect species, pathogenic, as well as free-living fungi (constructed on the base of sequences of Beta-tubulin gene). The numbers near the nodes refer to the Bayesian posterior probability. Table S1. List of primers and fluorochrome-labeled probe used in this study.
