# Supplementary material for: Fungal Associates of Soft Scale Insects (Coccomorpha: Coccidae)
Source: Cells. 2021 Jul 29;10(8):1922. doi: 10.3390/cells10081922 (PMC8394295; doi:10.3390/cells10081922)
Supplement: Supplementary file 1 [file cells-10-01922-s001.zip › Table S1.pdf]

Purpose	Primer name	Primer sequence (5'-3')	Target gene	Annealing temperature	Source
PCR	ITS3<br>ITS4	GCATCGATGAAGAACGCAGC<br>TCCTCCGCTTATTGATATGC	Fungal Internal Transcribed Spacer of nuclear ribosomal RNA (ITS2)	58°C	[49]
	Ophi_Btub44448F<br>Ophi_Btub5243R	CGyGAGGAGTTCCyGACCG<br>CrTCCTGGTAyTGCTGGTACTC	Beta-tubulin of *Ophiocordyceps* fungi	60°C	Vanderpool, unpublished
	PCoF1<br>HCO	CCTTCAACTAATCATAAAAATATyAG<br>TAAACTTCAGGGTGACCAAAAAATCA	Cytochrome c oxidase subunit I (COI)	51°C	[52]
FISH	HYP760	Cy5- CCTGCCTGGAGCACTCT	18S rRNA gene of *Ophiocordyceps* fungi	Not applicable	[53]

Table S1. List of primers and fluorochrome-labeled probe used in this study.
