# Supplementary figures and images for: Fungal Associates of Soft Scale Insects (Coccomorpha: Coccidae)
Source: Cells. 2021 Jul 29;10(8):1922. doi: 10.3390/cells10081922 (PMC8394295; doi:10.3390/cells10081922)

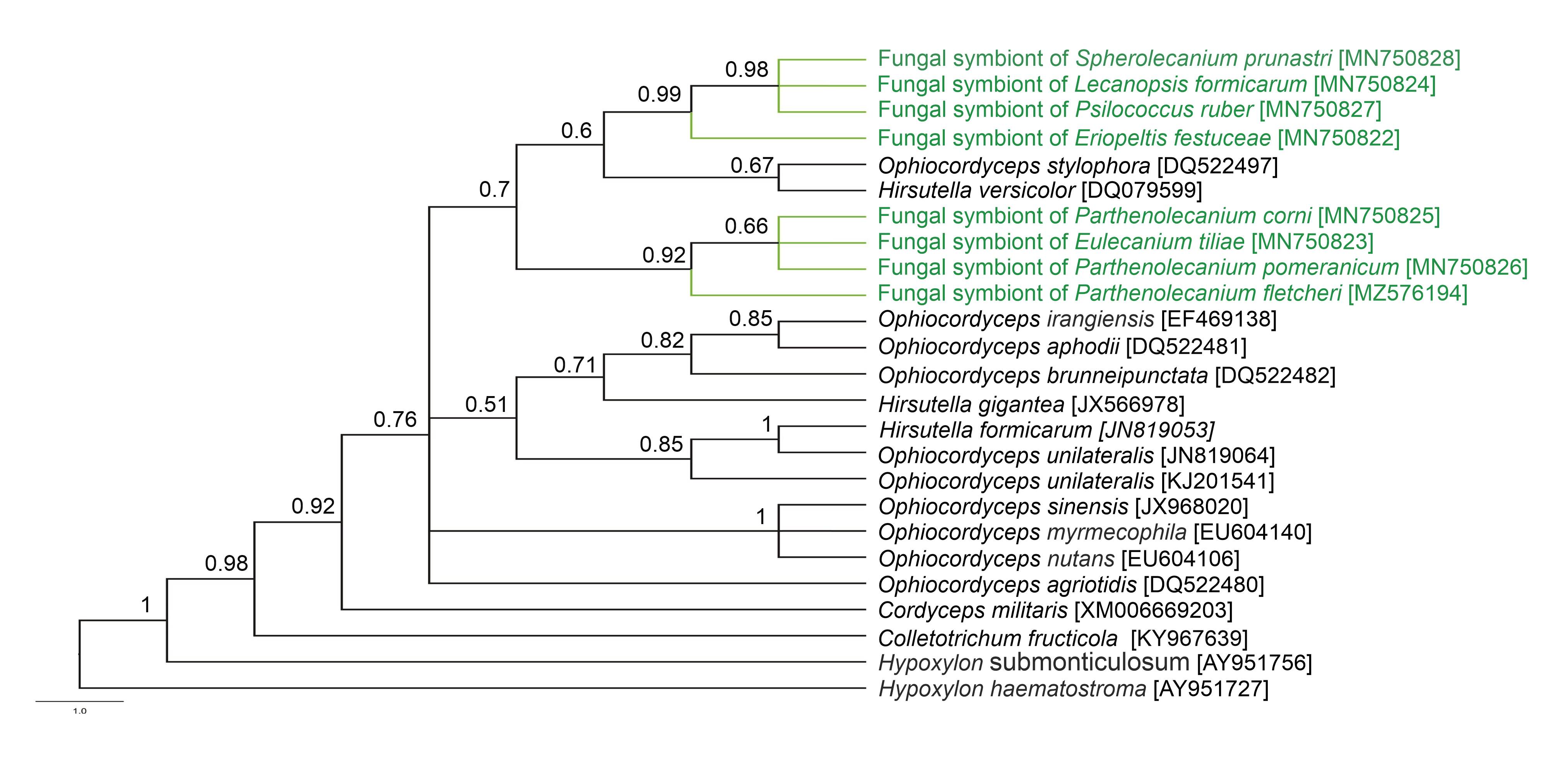

Supplement: Supplementary file 1 [file cells-10-01922-s001.zip › Figure S1.tif]
